# The impact of musical expertise and directional isotropy on the proportions and magnitudes of pitch-shift responses in glissandos

**DOI:** 10.3389/fpsyg.2024.1332028

**Published:** 2025-01-23

**Authors:** Li-Hsin Ning

**Affiliations:** Department of English, National Taiwan Normal University, Taipei City, Taiwan

**Keywords:** auditory perturbation, glissando, opposing response, following response, musical expertise

## Abstract

**Background:**

Previous studies have established that when vocal pitch in auditory feedback is perturbed unexpectedly, speakers typically produce opposing responses to correct the perceived error. Investigations comparing steady-pitch vocalizations and non-steady-pitch vocalizations have revealed that the extent of compensation is task-dependent. Nevertheless, the influence of musical expertise and the preference for adopting opposing or following responses during glissando vocalizations remain unexplored.

**Methods:**

In this study, thirty-six native Mandarin speakers, comprising equal numbers of musicians and non-musicians, were asked to perform three vocal tasks. During the sustained vowel task, participants maintained a steady and comfortable pitch while vocalizing /a/ for 3 s. In the upward glissando and downward glissando tasks, participants imitated the gliding pattern of the model note introduced at the beginning of each trial. The onset of pitch-shifted feedback (±100 cents) occurred randomly between 500 and 700 ms after vocal onset, lasting for 200 ms. Response proportions for opposing and following responses were estimated through Bayesian Poisson regression modeling, whereas response magnitudes were scrutinized using generalized additive mixed effects modeling.

**Results:**

Our results revealed that opposing and following responses were less pronounced among musicians compared to non-musicians. Furthermore, following responses were not a minority in response to auditory perturbations; rather, they constituted 42% of the responses on average. Additionally, response magnitudes were found to be contextually sensitive and were influenced by the direction of the shift and the intended pitch direction.

**Conclusion:**

Our results indicate that our ability to control vocal responses is influenced by context and that musicial training plays a role in affecting how participants react to auditory perturbations.

## 1 Introduction

Speech production is a complex motor skill that heavily relies on sensory feedback. Normal speech development in children, for instance, is critically dependent on auditory feedback. Individuals with post-lingual deafness typically experience immediate challenges in controlling pitch and loudness, while their articulatory intelligibility declines gradually (Perkell et al., 2000; Waldstein, 1989). The importance of auditory feedback has been well demonstrated through the altered auditory feedback paradigm across different acoustic domains (Bauer et al., 2006; Burnett et al., 1998; Houde and Jordan, 1998; Max et al., 2003). When pitch, loudness, or formant frequency in auditory feedback is altered either predictably or unpredictably, speakers typically adjust their voice in the opposite direction of the shift. The opposing response is termed “compensation” when adjusting to unpredictable perturbations and “adaptation” when adjusting to predictable perturbations. The opposing response suggests that speakers attempt to correct for the mismatch between the anticipated and perceived signals. In addition to the opposing response, speakers may also adjust their voice in the same direction as the shift, termed the “following response.” While the underlying mechanism of the following response remains uncertain, research has suggested that it may occur when the perturbation is viewed as an external reference (Hain et al., 2000), when larger perturbation magnitudes are used (Burnett et al., 1998), or when the perturbation direction is misperceived (Larson et al., 2007).

Previous research has indicated that the degree of compensation varies depending on the specific task. For instance, Chen et al. (2007) examined compensation in English speakers during sustained vowels (/u/) and English speech (“*you know Nina?*”). They found that compensation was more pronounced in the speech condition than in the sustained vowel condition (see Table 1 for more details). A task effect was also noted in Natke et al. (2003), where compensation was larger when singing (matching a specific note) the nonword [ˈta:tatas] compared to when speaking it. This suggests that compensation is increasingly pronounced as more precise pitch control is required. Furthermore, the task-specific effect emerged when considering compensation in either a sustained vowel (Liu and Larson, 2007; Sturgeon et al., 2015) or an English phrase (Liu et al., 2010) with different fundamental frequency (*f*_o_) values. Larger compensation was observed in the condition with higher *f*_o_ values compared to the condition with lower *f*_o_ values, indicating that audio-vocal control is influenced by the level of the laryngeal effort required.

**TABLE 1:** Summary of research using the altered auditory feedback paradigm.

References	Participants	Stimuli	Methodology	Results
Chen et al., 2007	English speakers	• Sustained vowel (/u/) • English speech (“you know Nina?”)	Unpredictable perturbations	Compensation: English speech > sustained vowel
Natke et al., 2003	German speakers	• Speaking the nonsense word [ˈta:tatas] with no reference pitch provided • Singing the nonsense word [ˈta:tatas] at a designated fixed pitch (females: 233 Hz, males: 123 Hz)	Unpredictable perturbations	Compensation: singing > speaking
Liu and Larson, 2007	English speakers	• Sustained vowel /u/ with a piano note close to average conversational *f*_o_ value Sustained vowel /u/ with a piano note at a level much higher than the average conversational *f*_o_.	Unpredictable perturbations	Compensation: high *f*_o_ voice > low *f*_o_ voice
Liu et al., 2010	English speakers	• English speech (“you know Nina?”) at a high *f*_o_ voice (300 Hz) • English speech (“you know Nina?”) at a low *f*_o_ voice (200 Hz)	Unpredictable perturbations	Compensation: high *f*_o_ voice > low *f*_o_ voice
Sturgeon et al., 2015	• English-speaking musical group: people who had taken vocal lessons outside of K-12 school or had private training on other instruments for more than 2 years • English-speaking non-musical group	• Sustained vowel /a/ at conversational *f*_o_ • Sustained vowel /a/ at 2 semitones below conversational *f*_o_ • Sustained vowel /a/ at 6 semitones above conversational *f*_o_	Unpredictable perturbations	Compensation: musical group > non-musical group; higher *f*_o_ voice > low *f*_o_ voice
Burnett and Larson, 2002	Trained singers	• Sustained vowel /u/ Upward glissando /u/	Unpredictable perturbations	Compensation: sustained vowel > upward glissando
Kim and Larson, 2019	• English-speaking musicians: people who had five or more years of musical instruction or continued practice • English-speaking non-musicians: people who did not have any musical instruction at least 3 years before	• Sustained vowel /ɑ/ with a steady *f*_o_ • Sustained vowel /ɑ/ with a raised *f*_o_	Unpredictable perturbations	Compensation: musicians > non-musicians; steady *f*_o_ = raised *f*_o_
Jones and Keough, 2008	• English-speaking trained singers • English-speaking non-singers	• Singing /ta/ at 392 Hz for the first 40 trials and then at 349 Hz for the remaining 20 trials • Singing /ta/ at 349 Hz or the first 40 trials and then at 392 Hz for the remaining 20 trials	Predictable perturbations	Adaptation: singers < non-singers
Ning, 2020	• Mandarin-speaking trained singers • Mandarin-speaking non-singers	• Sustained vowel /a/ • Mandarin tone word /ma1/ (“mother”) • Mandarin tone word /ma2/ (“hemp”)	Predictable perturbations	Adaptation: singers < non-singers; /ma2/ > /ma1/ > /a/
Zarate and Zatorre, 2005, 2008	• Trained singers • Non-singers	• Singing /a/ in blocks of five trials (each with different pitch: D#3, F3, G#3, B3, C#4 for males; an octave higher for females) and *ignoring* the shifted feedback • Singing /a/ in blocks of five trials and *compensating for* the shifted feedback	Unpredictable perturbations	Compensation in the ignore condition: singers < non-singers Compensation in the compensation condition: singers = non-singers

Several studies have investigated the impact of the musical expertise of trained singers and of trained instrument players on compensation and adaptation responses (Burnett and Larson, 2002; Jones and Keough, 2008; Kim and Larson, 2019; Ning, 2020; Sturgeon et al., 2015; Zarate and Zatorre, 2005, 2008). Beyond sustained vowels, speech tasks, and singing conditions, compensation has also been observed in the context of glissandos (Burnett and Larson, 2002). When compared to steady-pitch vocalizations, trained singers exhibited less compensation in the case of glissandos. This finding is inconsistent with the task effect suggested in the preceding paragraph, where pronounced compensation is observed when precise pitch control is required. Burnett and Larson’s (2002) study, however, left several questions unanswered. The first issue was the role of musical expertise, as their study included only singers. It is unclear whether less compensation for tasks requiring more precise pitch control is specific to singers.

The impact of musical expertise has yielded diverse findings in previous research. Kim and Larson (2019) and Sturgeon et al. (2015) observed that musicians (specifically instrument players) exhibited *greater* compensation than non-musicians. Conversely, Jones and Keough (2008) and Ning (2020) found an opposite pattern, with singers adapting *less* than non-singers in their studies. Zarate and Zatorre (2005, 2008) even reported that singers were capable of suppressing opposing responses to unpredictable perturbations (i.e., *reduced* compensation) and produced the target notes accurately.

This discrepancy between the two sets of findings may be influenced by several factors: the nature of musical expertise (singing or not), and the type of vocalization stimuli (any pitch, designated fixed pitch, or lexical tone). Singers often exhibit superior vocal control compared to instrument players, which could partly explain why Kim and Larson (2019) and Sturgeon et al. (2015) found greater compensation among musicians compared to non-musicians but Jones and Keough (2008), Ning (2020), and Zarate and Zatorre (2005) 2008 observed less adaptation/compensation when comparing singers to non-singers. On the other hand, in studies that identified *reduced* response magnitudes in singers compared to non-singers, participants were required to produce a designated fixed pitch value or a pitch contour that demanded more regulation, such as lexical tones (see Table 1 for more details) (Jones and Keough, 2008; Ning, 2020; Zarate and Zatorre, 2005, 2008). When producing a designated fixed pitch value that is not freely chosen by the vocalizer, or a lexical tone that involves a specific pitch pattern, the selection of the appropriate internal model of pitch to be activated may be more constrained. We speculate that this constraint may possibly make the vocalization task more demanding, allowing only trained singers to manage it without being significantly affected by auditory perturbations.

To explore the comprehensive effects of musical training, ideally, we would include three groups (singers, instrument players, non-musicians) in a single study. However, the relatively low admission rate in the vocalist track of the Music Department prevented us from finding a sufficient number of singers. Therefore, in this study, we focused on instrument players (referred to as musicians from now on) and non-musicians. Regarding the test stimuli, sustained vowels with a freely chosen pitch should require less precise knowledge of the internal representations for pitch than those with a designated pitch or vowels with a specific pitch contour. To cover both ends of the spectrum in terms of the preciseness of internal models, we decided to use sustained vowels with a freely chosen pitch and vowels with a specific changing pitch (glissandos). Given that glissandos involve precise pitch-changing information rather than random selection of pitch values seen in sustained vowels, musicians are expected to benefit from this precise pitch detail. We hypothesized that musicians should exhibit *reduced* response magnitudes rather than enhancement when compared to non-musicians (musicians < non-musicians), with this difference being more pronounced in the glissandos than in the sustained vowels (Hypothesis 1).

The second unresolved issue in Burnett and Larson (2002) pertains to the proportion of the following responses. Their claim of only 2 following responses out of 60 was derived from *averaged curves* rather than individual trials. However, a growing body of evidence reveals that following responses emerge when we analyze *trial-to-trial* response patterns. Following responses occurred at rates ranging from 45 to 56% in vowel production (Behroozmand et al., 2012; Franken et al., 2018; Korzyukov et al., 2012; Li et al., 2013), and from 35 to 50% in tone word production (Ning, 2022a). In this current study, we seek to extend the existing line of research by examining the proportions and magnitudes of following responses in the context of glissandos on a trial-to-trial basis.

In previous research, large compensation has been observed when there was a conflict between the pitch-shift direction and the intended rising pitch direction (Chen et al., 2007; Kim and Larson, 2019). Following responses, on the other hand, are likely to occur when perturbations are perceived as external references or when speakers unconsciously mimic the altered stimuli (Franken et al., 2018; Hain et al., 2000; Kim and Larson, 2019). We hypothesized that the frequency and magnitude of following responses observed on individual trials are also task-dependent, similar to opposing responses. Specifically, we predicted that following responses will be larger and more frequent (i) when the shift direction aligns with the glissando direction and (ii) during glissando vocalizations compared to steady-pitch vocalizations (Hypothesis 2). The conflict between pitch-shift direction and motor planning direction might lead speakers to believe their pitch is significantly different from the intended contour, prompting more compensation. Conversely, the alignment between pitch-shift direction and motor planning direction may reduce the degree of perceived mismatch, causing speakers to think they did not make a mistake and perceive the shifted pitch as originating from someone else, increasing the likelihood of observing following responses on a trial-to-trial basis.

Lastly, Burnett and Larson (2002) exclusively examined upward glissandos with down-shift stimuli, leaving uncertainty about the impact of downward glissandos and up-shift stimuli on vocal responses. In the present study, we explored both upward and downward glissandos, in addition to steady-pitch vowel vocalizations, combined with both upward and downward shifts. Previous research has suggested a directional effect, indicating that compensation tends to be more pronounced when the shift direction is opposite to the intended pitch (e.g., down-shifts in question intonation) (Chen et al., 2007; Kim and Larson, 2019). Thus, we hypothesized that a shift direction opposite to the glissando direction would result in a more substantial degree of compensatory responses (Hypothesis 3).

To sum up, this study aims to explore how musicians and non-musicians respond to auditory perturbations while producing sustained vowels, upward glissandos, and downward glissandos. The research will analyze the proportions and magnitudes of both opposing and following responses using Bayesian Poisson regression modeling and generalized additive mixed effect modeling.

## 2 Materials and methods

### 2.1 Participants

Thirty-six native Mandarin speakers were recruited to participate in the research. Half of them (9 males and 9 females; age range: 20–25 years; mean age: 21.33 years) were music majors and instrument players at the time of the experiment, proficient in piano, violin, cello, flute, tuba, or percussion. All the musicians have been playing their instruments for more than 12 years. The other half were non-musicians (9 males and 9 females; age range: 21–30 years; mean age: 25.16 years). Among the 18 non-musicians surveyed, 15 stated they have never learned to play a musical instrument outside of school curriculum. Two participants mentioned having learned piano during elementary school for less than 5 years, while another reported self-studying guitar for 1 year. Prior to the experiment, all participants underwent binaural hearing tests at frequencies of 250, 500, 750, 1000, 2000, 3000, and 4000 Hz using a MAICO pure-tone audiometer (model MA 25), with each ear tested separately. They all successfully passed the hearing screening test at 20 dB. They signed an informed consent approved by the institutional review board (Research Ethics Office) and received monetary compensation for their participation.

### 2.2 Procedure

Participants were instructed to vocalize the vowel /a/ in three different ways: sustained vowel (SVL), downward glissando (GDN), and upward glissando (GUP). In the sustained vowel (SVL) phonations, participants vocalized /a/ at a steady and comfortable pitch for 3 s, following a beep sound signaling the onset of a trial. For the glissandos (GDN and GUP), participants first listened to a model synthetic note and then were asked to imitate the gliding pattern in terms of its speed and duration, while staying within their own comfortable pitch range. The model note began with a steady tone for 500 ms (male: 100 Hz; female: 200 Hz), transitioned into either an upward or downward glide (100 cents/half second for 2 s), and concluded with another 500 ms of a steady note. In other words, the model note had a 400-cent difference between onset and offset pitches. In the sustained vowel (SVL) condition, no model note was provided as the purpose was to explore participants’ responses at a freely chosen pitch, representing one extreme (free and easy) compared to the other extreme where they had to model a specific rising and falling pattern (not free and potentially challenging). Therefore, the three conditions (GDN, GUP, and SVL) differed not only in terms of pitch pattern but also in terms of the presence or absence of imitation. Whether or not imitation is involved may be a confounding variable, which will be discussed in section “4.2 Response proportions are affected by the interaction between shift direction and intended pitch direction.”

Prior to recording, participants underwent a practice phase comprising 5 trials for each production condition. Within each vocalization, a pitch-shift stimulus of ±100 cents was presented, lasting for 200 ms. The onset of the pitch-shift stimuli could randomly occur between 500 and 700 ms after vocal onset. The pitch-shift stimuli could take the form of an upward shift, a downward shift, or no change (control), and they were equally likely to appear. Following the instructions used in the compensation studies by Burnett and Larson (2002) and Zarate and Zatorre (2005) 2008, participants were instructed to *ignore* the pitch-shift stimuli and maintain their intended pitch contour. The purpose of “ignoring perturbations” was to explore the involuntary control of pitch. Although the correct execution of such an instruction would be a “non-response” (i.e., no error correction, being it opposing or following), “non-responses” accounted for only 2% of the data, suggesting that pitch-shift responses (whether opposing or following) are automatic. It is this automaticity we aim to examine.

Each production condition (SVL, GDN, and GUP) consisted of 30 vocalizations, resulting in a total of 90 trials (30×3). The inter-trial delay was 1000 ms. The order of the three production conditions was randomized across participants. The entire experiment took approximately 30 min.

### 2.3 Apparatus

Participants sat inside a soundproof booth and wore AKG K240 headphones. In front of them, we placed an Audio Tech ATR20 standalone microphone, positioned 2 cm away from their mouths. The microphone’s voice signal underwent real-time pitch shift, with a delay of approximately 14–20 ms (measured by the latency difference between the microphone and headphone channels). The real-time pitch shift was facilitated by an Eventide Ultra-Harmonizer (model H7600), which was controlled using Max/MSP (version 7, developed by Cycling). To mask bone-conducted auditory feedback, we boosted the voice signal by 10 dB using a McLELLAND MAR-16P headphone amplifier when it was played back through the headphones. The intensities of the microphone and the headphone signals were calibrated using a BENETECH digital sound level meter (GM1351). The microphone level was calibrated to 77 dB and the headphone signal was set to 87 dB (with a 10 dB gain). We placed the sound level meter in front of the participants to allow them to monitor their voice volume during vocalizations. The participants were asked to maintain a loudness level of 77 ± 2 dB. The WinDaq DI-720 acquisition device was used to record the vocalizations, the pitch-shifted signals, and the TTL pulses that indicated the onset of pitch-shift stimuli. These signals were sampled at a rate of 8 kHz per channel using WinDaq Pro.

### 2.4 Data preprocessing

The signals recorded in WinDaq Pro were imported into MATLAB (R2020a) and sorted based on the direction of pitch-shift stimuli (up-shift, down-shift, and control). Each vocalization was segmented into a 1200 ms-long signal, encompassing a 200 ms pre-shift period, a 200 ms shift period, and an 800 ms post-shift period. These segmented voice signals were then converted into sound files and processed in Praat to estimate pitch values at 10 ms intervals. The pitch values were then imported back to MATLAB and transformed into cents using the formula: cents = 1200 × log2(*f*_o_/baseline), where the baseline represented the mean pitch of the pre-shift period.

Each segmented vocalization was categorized into one of four response types: opposing response, following response, non-response, and error. This categorization relied on visual aid of the pre-shift mean and confidence intervals. In the case of pitch contours in the SVL condition, the confidence intervals encompassed two standard deviations of the pre-shift curves in each individual trial. For pitch contours in the GDN and GUP conditions, regression lines with 95% confidence intervals were generated by fitting them to the pre-shift period and then extending them across the entire curve. During the classification process, the response was evaluated by comparing it to the pre-shift period. If the response exhibited a change in the opposite direction to the pitch-shift stimulus and exceeded the confidence intervals, it was categorized as an “opposing” response. Conversely, if the response changed in the same direction as the pitch-shift stimulus and surpassed the confidence intervals, it was categorized as a “following” response. A “non-response” label was applied when the response did not clearly deviate upward or downward from the averaged control, remaining within the bounds of the confidence intervals. Lastly, if the response yielded an erroneous pitch-tracking result, it was designated as an “error.”

After the classification, we computed the percentage of opposing responses, following responses, non-responses, and errors for each participant under each condition (3 production conditions × 2 pitch-shift directions) by dividing the raw counts by the total counts within each condition. On average, across conditions, opposing responses were 50% and following responses were 42%. Non-responses (2%) and errors (6%) were subsequently excluded from further analysis. The voices of two male non-musicians could not be accurately estimated by Praat. Consequently, their data were entirely excluded from the statistical analysis. Difference waves were obtained for the sustained vowels, the upward glissando, and downward glissando by subtracting the averaged pitch contour of the corresponding control trials from the averaged opposing or following pitch contours at every data point.

### 2.5 Statistical analyses

We utilized Bayesian Poisson regressions to estimate the proportional differences in opposing responses and following responses, considering musical expertise, production, and pitch-shift direction as conditioning factors. For the pitch response contours, we employed generalized additive mixed effect models to assess the difference waves (in cent values) for the upward and downward glissando conditions, as well as the standardized pitch contours (in cent values) for the steady-vowel condition.

## 3 Results

The participants produced an average difference of 389 cents (SD = 13) for upward glissandos and 375 cents (SD = 7) for downward glissandos, both of which are very close to the model note (400 cents). The results regarding the proportions of opposing and following responses, along with their response contours, are presented below.

### 3.1 The proportions of opposing and following responses

The brms package (Bürkner, 2017) in R (R Core Team, 2022) was employed to predict response proportions with respect to musical expertise (non-musicians and musicians), production (SVL, GDN, and GUP), pitch-shift direction (down and up), and response type (opposing and following). Given the nature of count data, the poisson family function was used. Non-informative priors were applied to maximize the data’s impact. To simulate samples, four Markov chains with 4000 iterations per chain were employed, with 1000 samples allocated for warmup. The inclusion of a random intercept, (1| participant), did not yield a better fit (LOOIC = 8661 compared to LOOIC = 8404); therefore, we retained the original model without the random intercept.

Supplementary Figure 1 illustrates trace plots of parameter draws obtained from Markov chain Monte Carlo (MCMC) simulations across the four chains. The overlapping batches of time series data indicate successful convergence for all chains. Additional convergence diagnostics, including $\hat{R}$ (the potential scale reduction factor on split chains), bulk effective sample size (Bulk ESS), and tail effective sample size (Tail ESS), are provided in Table 2. An $\hat{R}$ value of 1 and an effective sample size exceeding 1000 suggest that the drawn samples have achieved convergence.

**TABLE 2:** Summary table of the Bayesian Poisson regression model (PROPORTION ∼ MUSICAL EXPERTISE × PRODUCTION × DIRECTION × RESPTYPE).

Parameter		Estimate	Est. error	Lower-95% CI	Upper-95% CI	Rhat	Bulk ESS	Tail ESS
Intercept		3.87	0.04	3.80	3.95	1.00	3845	5840
MUSICAL EXPERTISE	Musician	0.16	0.05	0.07	0.26	1.00	3730	5785
PRODUCTION	GDN	0.18	0.05	0.08	0.28	1.00	4076	6662
	GUP	−0.32	0.06	−0.44	−0.21	1.00	4097	6606
DIRECTION	Up	0.08	0.05	−0.03	0.18	1.00	3695	6180
RESPTYPE	Following	−0.09	0.05	−0.20	0.01	1.00	3690	6228
MUSICAL EXPERTISE × PRODUCTION	Musician × GDN	−0.33	0.07	−0.47	−0.20	1.00	4032	6747
	Musician × GUP	−0.16	0.08	−0.30	−0.01	1.00	4016	6091
MUSICAL EXPERTISE × DIRECTION	Musician × Up	−0.15	0.07	−0.28	−0.02	1.00	3715	5820
PRODUCTION × DIRECTION	GDN × Up	−0.56	0.08	−0.70	−0.41	1.00	4037	6255
	GUP × Up	0.47	0.08	0.32	0.62	1.00	3952	6203
MUSICAL EXPERTISE × RESPTYPE	Musician × Following	−0.25	0.07	−0.39	−0.11	1.00	3684	6006
PRODUCTION × RESPTYPE	GDN × Following	−0.72	0.08	−0.88	−0.56	1.00	4030	6036
	GUP × Following	0.56	0.08	0.40	0.71	1.00	3886	6268
DIRECTION × RESPTYPE	Up × Following	−0.08	0.08	−0.23	0.06	1.00	3641	5894
MUSICAL EXPERTISE × PRODUCTION × DIRECTION	Musician × GDN × Up	0.68	0.10	0.49	0.87	1.00	4007	6229
	Musician × GUP × Up	0.01	0.10	−0.19	0.21	1.00	4015	5909
MUSICAL EXPERTISE × PRODUCTION × RESPTYPE	Musician × GDN × Following	0.94	0.11	0.73	1.15	1.00	4004	6342
	Musician × GUP × Following	0.22	0.10	0.02	0.43	1.00	3819	6393
MUSICAL EXPERTISE × DIRECTION × RESPTYPE	Musician × Up × Following	0.25	0.10	0.05	0.45	1.00	3687	6034
PRODUCTION × DIRECTION × RESPTYPE	GDN × Up × Following	0.99	0.12	0.77	1.22	1.00	3932	6617
	GUP × Up × Following	−1.36	0.11	−1.58	−1.14	1.00	4184	5948
MUSICAL EXPERTISE × PRODUCTION × DIRECTION × RESPTYPE	Musician × GDN × Up × Following	−1.27	0.15	−1.56	−0.97	1.00	3972	6518
	Musician × GUP × Up × Following	0.52	0.15	0.23	0.81	1.00	4119	6220

Shaded cells indicate cases where the 95% credible intervals encompass zero, indicating insignificance.

Table 2 and Supplementary Figure 2 present the posterior distributions of parameter draws, including estimates, standard errors of the mean, 95% credible intervals. These distributions reveal that there were no significant main effects of pitch-shift direction and response type, and no significant interaction between them, as evidenced by the 95% credible intervals encompassing zero.

**FIGURE 1:**
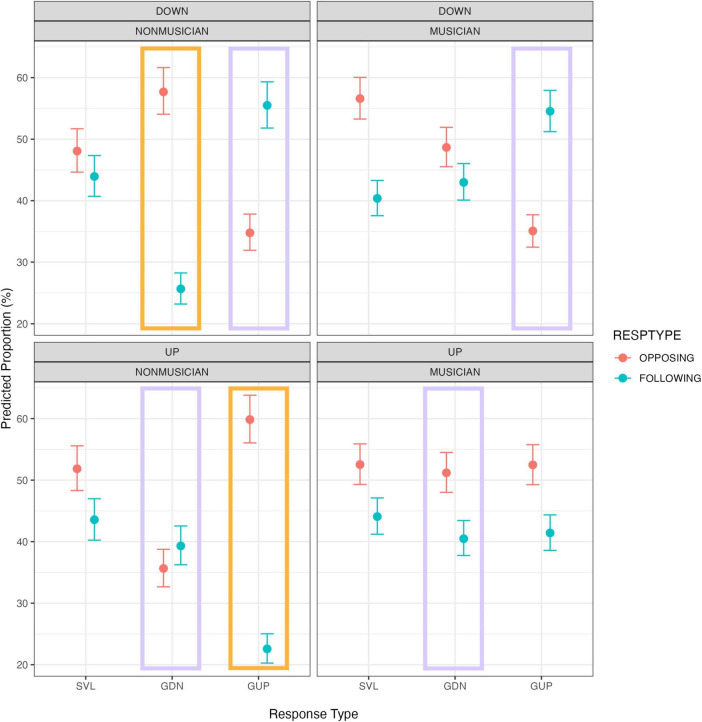
Conditional plots for the interaction of production and response type conditioned on musical expertise and pitch-shift direction. The proportion data represent predictive values obtained from posterior distributions. The bars depict the 95% credible intervals. The orange rectangles indicate nonmusicians’ tendency to oppose rather than follow when the pitch-shift direction matched the intended pitch contour. The purple rectangles indicate cases when the pitch-shift direction opposed the glissando direction.

Significant interactions can be visualized in Figure 1. Generally, in the SVL condition, both non-musicians and musicians exhibited a tendency to favor opposing responses over following responses, irrespective of pitch-shift direction (down or up). When it came to glissandos, non-musicians displayed a notably higher preference for opposing responses than following responses when the pitch-shift direction matched the intended pitch contour (i.e., a down-shift in GDN and an up-shift in GUP). However, this preference for opposing responses over following responses was less pronounced among musicians. Interestingly, for both non-musicians and musicians, following responses became more prevalent than opposing responses when they responded to down-shift stimuli in GUP. These patterns observed in the context of glissandos suggest that participants tended to reduce the pitch slope of glissandos when pitch perturbation occurred in the auditory feedback.

### 3.2 The response contours

The bam() function from the mgcv package (Wood, 2021) in R was used to model the time series data for opposing responses and following responses separately. To compare upward-going and downward-going pitch responses, absolute pitch values in cents were used as the dependent variable. We employed the generalized additive mixed effect models (GAMMs) to assess the effect of musical expertise (non-musicians and musicians), production (SVL, GDN, and GUP), and pitch-shift direction (down and up) individually with the following specifications:

1. A smooth term s(TIME) and a factor smooth s(TIME, SUBJECT, bs = “fs”) were included to capture time-varying differences and individual nonlinear variability.
2. A parameter rho was used to estimate the auto-correlated residuals within the time series data.
3. The scaled-*t* family distribution was applied due to the heavy-tailed nature of the data.

Non-musicians, SVL, and down-shifts were chosen as the reference levels for each variable. To facilitate pairwise comparisons, we created ordered factors for all combinations of musical expertise, production, and pitch-shift direction (2 × 3 × 2 = 12 levels), except for the reference level. For more comprehensive information on setting up the ordered factors, please refer to Wieling (2018), Sun and Shih (2021), and Ning (2022a).

**FIGURE 2:**
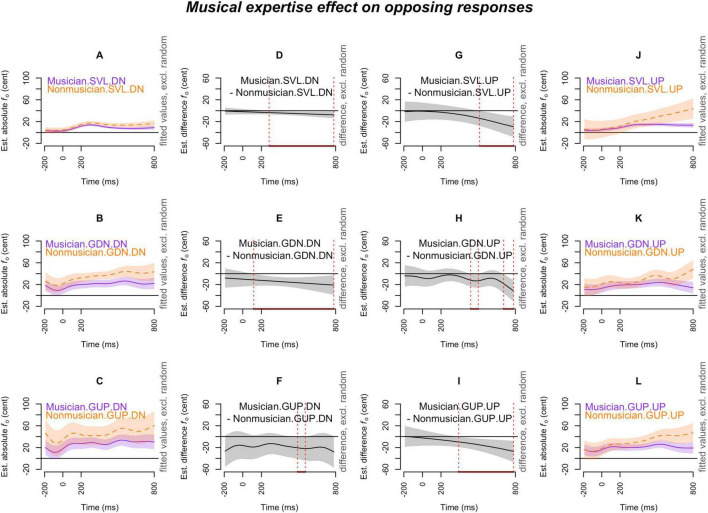
Musical expertise effect (musicians vs. non-musicians) on *opposing responses* across production types (SVL, GDN, and GUP), and pitch-shift directions (down and up). The *x*-axis ticks at 0 and 200 indicate the onset and offset of pitch-shift stimuli. Subplots **(A–C)** display the nonlinear change over time in the absolute fundamental frequency (*f*_o_) contours for musicians (purple curve) and non-musicians (orange curve) in the down-shifts of each production. Subplots **(D–F)** presents the difference waves between musicians and non-musicians in the down-shifts of each production. Subplots **(G–I)** depict the difference waves between musicians and non-musicians in the up-shifts of each production. Subplots **(J–L)** display the nonlinear change over time in the absolute *f*_o_ contours for musicians (purple curve) and non-musicians (orange curve) in the up-shifts of each production. The red line on the *x*-axis and the vertical dotted lines in the middle two columns indicate the time points at which the difference between musicians and non-musicians significantly deviates from zero.

**FIGURE 3:**
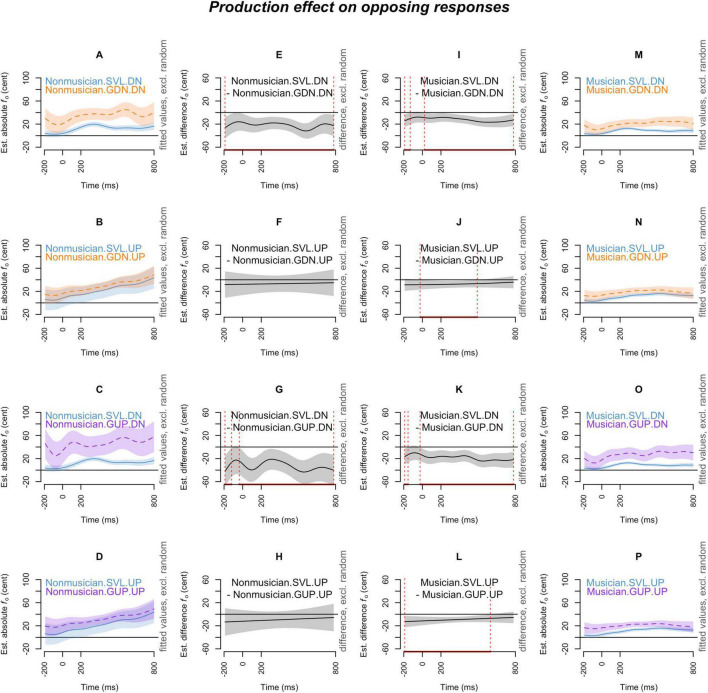
Production effect (SVL, GDN, and GUP) on *opposing responses* across musical expertise levels (musicians and non-musicians), and pitch-shift directions (down and up). The *x*-axis ticks at 0 and 200 indicate the onset and offset of pitch-shift stimuli. Subplots **(A–D)** display the nonlinear change over time in the absolute fundamental frequency (*f*_o_) contours for non-musicians in the SVL (blue curve), GDN (orange curve), and GUP (purple curve) conditions for each pitch-shift direction. Subplots **(E–H)** present the difference waves between sustained vowels and downward glissandos, as well as between sustained vowels and upward glissandos, for non-musicians in each pitch-shift direction. Subplots **(I–L)** depict the difference waves for musicians between sustained vowels and downward glissandos, and between sustained vowels and upward glissandos, in each pitch-shift direction. Subplots **(M–P)** display the nonlinear change over time in the absolute fundamental frequency (*f*_o_) contours for musicians in the SVL (blue curve), GDN (orange curve), and GUP (purple curve) conditions in each pitch-shift direction. The red line on the *x*-axis and the vertical dotted lines in the middle two columns indicate the time points at which the difference between production types significantly deviates from zero.

**FIGURE 4:**
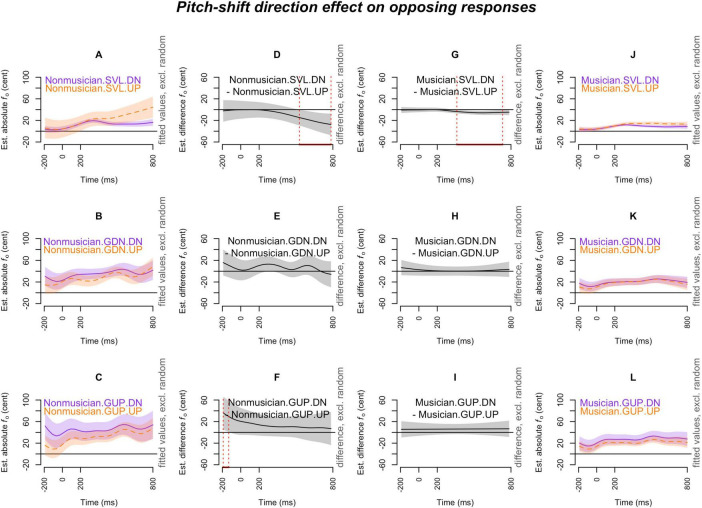
Pitch-shift direction effect (down vs. up) on *opposing responses* across production types (SVL, GDN, and GUP), and musical expertise levels (musicians and non-musicians). The *x*-axis ticks at 0 and 200 indicate the onset and offset of pitch-shift stimuli. Subplots **(A–C)** display the nonlinear change over time in the absolute fundamental frequency (*f*_o_) contours for down-shifts (purple curve) and up-shifts (orange curve) in each production type for non-musicians. Subplots **(D–F)** present the difference waves between down-shifts and up-shifts for non-musicians across each production type. Subplots **(G–I)** depict the difference waves for musicians between down-shifts and up-shifts in each production type. Subplots **(J–L)** display the nonlinear change over time in the absolute fundamental frequency (*f*_o_) contours for down-shifts (purple curve) and up-shifts (orange curve) for musicians in each production type. The red line on the *x*-axis and the vertical dotted lines in the middle two columns indicate the time points at which the difference between down-shifts and up-shifts significantly deviates from zero.

**FIGURE 5:**
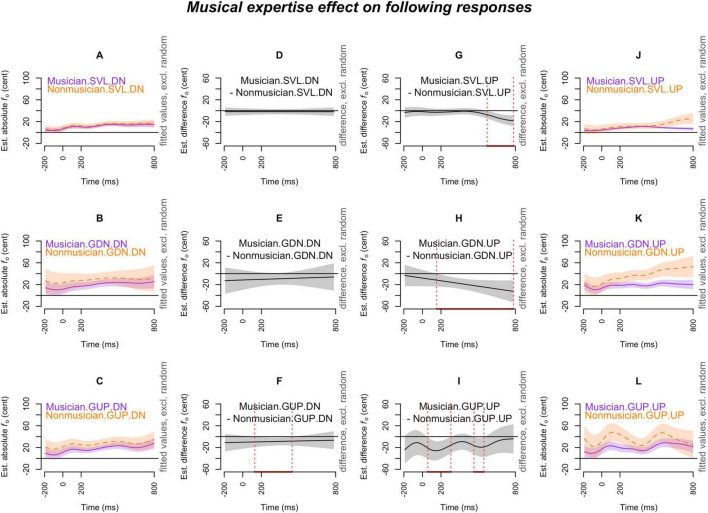
Musical expertise effect (musicians vs. non-musicians) on *following responses* across production types (SVL, GDN, and GUP), and pitch-shift directions (down and up). The *x*-axis ticks at 0 and 200 indicate the onset and offset of pitch-shift stimuli. Subplots **(A–C)** display the nonlinear change over time in the absolute fundamental frequency (*f*_o_) contours for musicians (purple curve) and non-musicians (orange curve) in the down-shifts of each production. Subplots **(D–F)** present the difference waves between musicians and non-musicians in the down-shifts of each production. Subplots **(G–I)** depict the difference waves between musicians and non-musicians in the up-shifts of each production. Subplots **(J–L)** display the nonlinear change over time in the absolute *f*_o_ contours for musicians (purple curve) and non-musicians (orange curve) in the up-shifts of each production. The red line on the *x*-axis and the vertical dotted lines in the middle two columns indicate the time points at which the difference between musicians and non-musicians significantly deviates from zero.

**FIGURE 6:**
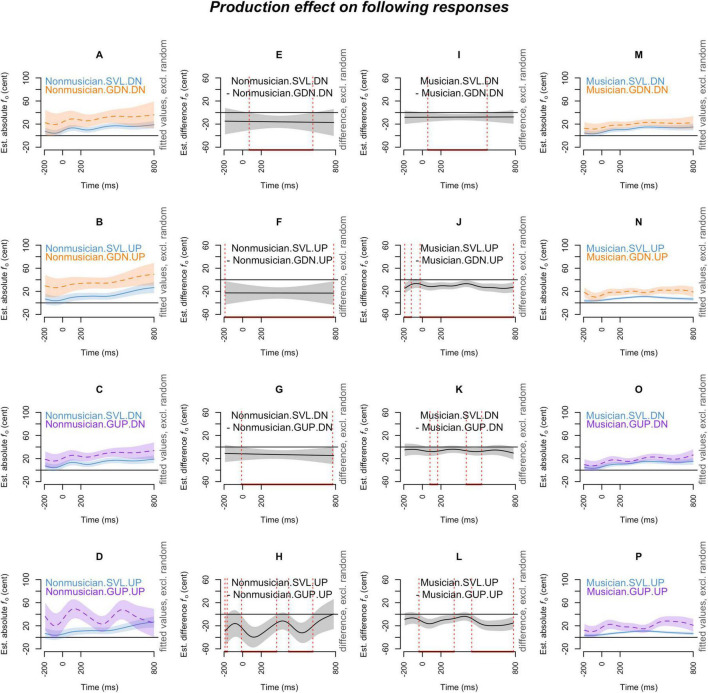
Production effect (SVL, GDN, and GUP) on *following responses* across musical expertise levels (musicians and non-musicians), and pitch-shift directions (down and up). The *x*-axis ticks at 0 and 200 indicate the onset and offset of pitch-shift stimuli. Subplots **(A–D)** display the nonlinear change over time in the absolute fundamental frequency (*f*_o_) contours for non-musicians in the SVL (blue curve), GDN (orange curve), and GUP (purple curve) conditions for each pitch-shift direction. Subplots **(E–H)** present the difference waves between sustained vowels and downward glissandos, as well as between sustained vowels and upward glissandos, for non-musicians in each pitch-shift direction. Subplots **(I–L)** depict the difference waves for musicians between sustained vowels and downward glissandos, and between sustained vowels and upward glissandos, in each pitch-shift direction. Subplots **(M–P)** display the nonlinear change over time in the absolute fundamental frequency (*f*_o_) contours for musicians in the SVL (blue curve), GDN (orange curve), and GUP (purple curve) conditions in each pitch-shift direction. The red line on the *x*-axis and the vertical dotted lines in the middle two columns indicate the time points at which the difference between production types significantly deviates from zero.

**FIGURE 7:**
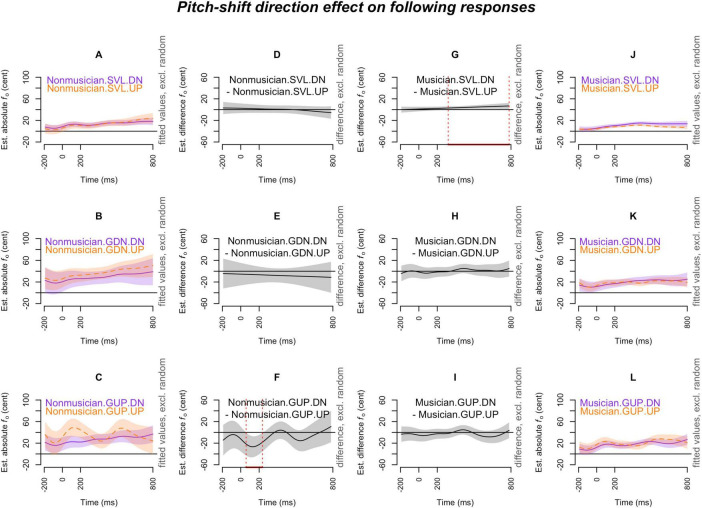
Pitch-shift direction effect (down vs. up) on *following responses* across production types (SVL, GDN, and GUP), and musical expertise levels (musicians and non-musicians). The *x*-axis ticks at 0 and 200 indicate the onset and offset of pitch-shift stimuli. Subplots **(A–C)** display the nonlinear change over time in the absolute fundamental frequency (*f*_o_) contours for down-shifts (purple curve) and up-shifts (orange curve) in each production type for non-musicians. Subplots **(D–F)** present the difference waves between down-shifts and up-shifts for non-musicians across each production type. Subplots **(G–I)** depict the difference waves for musicians between down-shifts and up-shifts in each production type. Subplots **(J–L)** display the nonlinear change over time in the absolute fundamental frequency (*f*_o_) contours for down-shifts (purple curve) and up-shifts (orange curve) for musicians in each production type. The red line on the *x*-axis and the vertical dotted lines in the middle two columns indicate the time points at which the difference between down-shifts and up-shifts significantly deviates from zero.

Figures 2–7 showed the smoothed curves simulated from the generalized additive models. To interpret the graphs, we start by examining the individual curves of each condition in the leftmost and rightmost columns. To estimate the differences between the curves in each subplot, difference waves were generated and plotted in the middle two columns (middle left column for the leftmost subplots and middle right column for the rightmost subplots), with the dotted red lines indicating regions of significant difference.

#### 3.2.1 Opposing responses

Figure 2 illustrates the musical expertise effect on opposing responses. As shown in Figures 2A–C, J–L, musicians exhibited smaller opposing curves than non-musicians across all production scenarios, regardless of pitch-shift direction (**musicians < non-musicians**). This distinction emerged as early as 100 ms following the onset of the pitch-shift stimulus (see Figure 2E) and could persist throughout the analysis window (see Figures 2D, E, G, I).

Figure 3 demonstrates the effect of production type on opposing responses. In Figures 3A–D, *non-musicians* generally exhibited larger opposing responses in the glissandos than in the sustained vowels. However, this difference (**glissandos > sustained vowels**) in opposing responses for non-musicians was only significant in the down-shift condition (Figures 3E, G). Similarly, as shown in Figures 3M–P, *musicians’* opposing responses in the glissandos were larger than their opposing responses in the sustained vowels, with the contrast (glissandos > sustained vowels) being significant and more prominent in the down-shift condition (Figures 3I, K) than in the up-shift condition (Figures 3J, L). Overall, both musicians and non-musicians exhibited significantly greater *pitch-increasing* responses (as opposing responses to down-shift stimuli) in the glissandos compared to sustained vowels (**glissandos > sustained vowels**), with this difference being even more pronounced in the GUP condition than in the GDN condition (as seen in the first and the third rows of Figure 3).

Figure 4 depicts the pitch-shift direction effect on opposing responses. Both musicians (Figures 4A, D) and non-musicians (Figures 4G, E) exhibited significantly larger opposing responses to up-shift stimuli compared to down-shift stimuli (**up-shift > down-shift**) in the SVL condition, meaning that *pitch-decreasing* responses were more pronounced than pitch-increasing responses in the SVL condition. However, this directional distinction was completely absent in the context of glissando productions (the mid and bottom rows of Figure 4).

#### 3.2.2 Following responses

Figure 5 demonstrates the influence of musical expertise on the following responses. In Figures 5J–L, musicians consistently displayed smaller following responses than non-musicians in all production scenarios involving up-shift stimuli (**musicians < non-musicians**). This difference was significant at the late stage for sustained vowels (Figure 5G) but at the early stage for glissandos (Figures 5H, I). The distinction between musicians and non-musicians (**musicians < non-musicians**) was also evident in the upward glissandos (GUP) with down-shift stimuli (Figures 5C, F), but not in the case of sustained vowels (SVL) with down-shift stimuli (Figures 5A, D) and downward glissandos (GDN) with down-shift stimuli (Figures 5B, E).

The production effect on the following responses is depicted in Figure 6. Similar to the pattern observed in opposing responses, both musicians (Figures 6M–P) and non-musicians (Figures 6A–D) exhibited significantly larger following responses in the glissandos than their following responses in the sustained vowels. However, contrary to the opposing responses, this contrast (glissandos > sustained vowels) in the following responses was more pronounced in the up-shift condition (Figures 6F, J, H, L) than in the down-shift condition (Figures 6E, I, G, K). These findings suggest that both musicians and non-musicians displayed significantly greater *pitch-increasing* responses (as following responses to up-shift stimuli) in the glissandos compared to sustained vowels (glissandos > sustained vowels), with this difference being even more pronounced in the GUP condition than in the GDN condition (as seen in the second and the fourth rows of Figure 6).

Figure 7 illustrates the pitch-shift direction effect on the following responses. The only significant findings were observed in musicians’ SVL condition (down-shift > up-shift; Figures 7G, J) and non-musicians’ GUP condition (up-shift > down-shift; Figures 7C, F). In the musicians’ SVL condition, *pitch-decreasing* responses (i.e., following the down-shift stimuli) were more pronounced than pitch-increasing responses. Conversely, an opposite pattern was observed in non-musicians’ GUP condition, where *pitch-increasing* responses (i.e., following the up-shift stimuli) were more prominent than pitch-decreasing responses.

## 4 Discussion

In this study, we aimed to examine how musicians and non-musicians respond to pitch-shifted stimuli in auditory feedback when producing sustained vowels, upward glissandos, and downward glissandos. The primary outcome measures of interest were the response proportions and response magnitudes. Response proportions were estimated using Bayesian Poisson regression modeling whereas response magnitudes were assessed through generalized additive mixed effects modeling.

### 4.1 Musicians are less susceptible to auditory perturbations

Our Bayesian Poisson regression results revealed that the distributions of opposing and following responses were relatively *similar* between musicians and non-musicians (see Figure 1). A slight difference was observed (see the orange rectangles in Figure 1): non-musicians exhibited a higher proportion of opposing responses than following responses when the pitch-shift direction matched the intended pitch contour, while musicians had more balanced distributions between opposing and following responses. Previous research has linked enhanced opposing responses with a greater reliance on auditory feedback (Jones and Keough, 2008; Liu et al., 2010; Scheerer and Jones, 2012). This scenario may occur when speakers vocalize at a high pitch (Liu et al., 2010), or in non-singers (Jones and Keough, 2008; Scheerer and Jones, 2012). Although opposing responses were the majority in most conditions for both musicians and non-musicians in the current study, the tendency to oppose rather than follow when the pitch-shift direction matched the intended pitch contour (as shown by the orange rectangles in Figure 1) indicates that the non-musicians still relied more on auditory feedback than musicians did.

In terms of the response magnitudes, musicians in general exhibited reduced opposing responses and following responses compared to non-musicians. Our first hypothesis that musicians should exhibit reduced response magnitudes, regardless of whether in opposing or following responses, rather than enhancement when compared to non-musicians was supported. These findings align with previous research by Jones and Keough (2008), Ning (2020), and Zarate and Zatorre (2005) 2008, but were inconsistent with Kim and Larson (2019) and Sturgeon et al. (2015), who argued that musicians exhibited *greater* compensation than non-musicians.

In the introduction, we argue that the distinct findings—reduced or enhanced responses in speakers with musical training—may be due to the nature of musical expertise (whether one sings or not) or the type of vocalization stimuli (any pitch, fixed pitch, or lexical tone). In the current study, the musicians exhibited reduced response magnitudes in both sustained vowels and glissandos (Figures 2D–I, 5F–I), suggesting that using any pitch or fixed pitch pattern did not matter. Since no singers were included, we could not examine the nature of musical expertise (i.e., singers vs. musicians). One methodological limitation of the current study is that participants were instructed to ignore pitch-shifted feedback, whereas in Kim and Larson (2019) and Sturgeon et al. (2015), no specific instructions were given on how to respond to auditory perturbations. Sturgeon et al. (2015) observed larger pitch-shift responses in musicians compared to non-musicians, with the difference between the two groups becoming more pronounced when the goal was to maintain a target high pitch. The reduction observed in our musicians may be due to their ability, developed through musical training, to block out errors in the environment (auditory perturbations) compared to non-musicians, or because our participants vocalized at a comfortable pitch (rather than a high pitch). To assess the ability to ignore errors, several approaches could be explored in future studies. For example, as 100-cent shifts are typically detectable by non-musicians, future research could examine smaller pitch shifts (e.g., 10 or 50 cents), making participants unaware of the change, so they would not know to ignore it. Alternatively, studies could include different instructions, such as “to compensate,” or provide no specific instruction at all. These approaches could help clarify the role of awareness and instruction in compensation to pitch shifts.

### 4.2 Response proportions are affected by the interaction between shift direction and intended pitch direction

Our second hypothesis was concerned with the proportions and magnitudes of following responses. It is evident that on a trial-to-trial basis, across both groups and all conditions, following responses constitute 42% of vocal responses, hardly a small minority of responses. However, contrary to our expectations, a significant prevalence of following responses over opposing responses (following > opposing) was observed solely when the pitch-shift direction went in the *opposite* direction compared to the glissando direction, with the exception of musicians’ up-shifts in GDN (see the purple rectangles in Figure 1). On the other hand, more opposing responses than following responses (opposing > following) were found in the glissandos with isotropic pitch-shifts, as well as in the sustained vowels. This weighting between opposing responses and following responses in glissandos (such as *following* the downshifts in the upward glissandos or *opposing* the downshifts in the downward glissandos) suggests that our participants tended to decrease the pitch slope. We suspect that the slope reduction may be associated with the real-time calculation of pitch contour adjustments in response to pitch perturbations. Participants might become conservative in raising or decreasing their pitch gradually when auditory perturbations appear.

Furthermore, in contrast to our second hypothesis, the occurrence of following responses in glissandos was no higher than in the sustained vowels. This pattern was inconsistent with the findings of Chen et al. (2007), where they observed a higher frequency of following responses in speech tasks compared to sustained vowel tasks. It is worth noting that their observations were based on averaged curves rather than individual pitch contours. It appears that on a trial-to-trial basis, the task-dependent effect on *response proportions* may diminish; instead, response proportions may be influenced by factors such as musical expertise and the interaction between shift direction and intended pitch direction addressed in the previous paragraph.

Section “2.2 Procedure” has identified that whether or not imitation occurs could be a confounding variable, as imitation was involved in glissandos but not in sustained vowels. Although the underlying mechanism of following responses remains unclear, following responses have been associated with perceiving pitch-shift stimuli as coming from someone else’s voice (Hain et al., 2000; Kim and Larson, 2019), misidentifying the direction of pitch-shift stimuli (Franken et al., 2018), or unconsciously mimicking the altered stimuli (Behroozmand et al., 2012). If imitation plays an essential role, we would likely observe more following responses than opposing responses in the glissandos but not in the sustained vowels. However, this tendency only occurred in the down-shifts of upward glissandos but not across all glissando conditions. We remain uncertain whether providing a model note would change participants’ responses in the sustained vowels. Imitation also raises another issue: whether to imitate the target pitch value or the pitch pattern. Since the focus of the current study was to compare pitch patterns (steady pitch vs. gliding pitch), providing a model note may further confuse participants about whether they should match the model pitch or simply sustain their own pitch. To assess the effect of imitation, future research may have to consider the interactions among imitation (presence or absence), target pitch value (matched or not), and the pitch pattern (steady or gliding).

### 4.3 The magnitudes of pitch-shift responses are task-dependent and sensitive to shift direction

The results of generalized additive mixed effects modeling indicate that pitch-shift responses, including both opposing and following, were significantly larger in the glissandos compared to the sustained vowels (**glissandos > sustained vowels**) when the pitch adjustments in voice manifested an *upward-going* direction, such as opposing a down-shift (Figures 3E, G, I, K) and following an up-shift (Figures 6F, H, J, L). However, within sustained vowels (in the SVL condition), pitch-decreasing responses, such as opposing an up-shift (Figures 4D, G) and following a down-shift (Figure 7G), were more pronounced than pitch-increasing responses; this pattern was consistently observed in musicians’ sustained vowels but not in non-musicians’ sustained vowels (Figure 7D). In other words, the effects of task dependency and pitch-shift direction on *response magnitudes* are more complicated than what was anticipated by our second and third hypotheses. The distinction between steady pitch and raised pitch has also been observed by Liu et al. (2009). They examined a four-word sentence uttered with a question intonation or a statement intonation and found that compensation was sensitive to the planning stage in the question intonation but not in the statement intonation. The difference between glissandos and sustained vowels, as found in the present study, suggests that the regulation of steady pitch and non-steady pitch may involve different motoric plans (pronounced pitch-increasing responses in glissandos or pronounced pitch-decreasing responses in sustained vowels) in our auditory-motor system.

In previous studies, such as Chen et al. (2007) and Kim and Larson (2019), it was observed that compensations for downward perturbations resulted in larger response magnitudes compared to upward perturbations. This directional effect was evident when a raised pitch was required in the utterance and the large compensation was associated with the conflict between the pitch-shift direction and the intended pitch direction. In our present study, unlike previous studies where following responses were excluded from the analyses, we considered both opposing responses and following responses. This comprehensive approach led to the finding that the degree of *response magnitudes* is influenced by both the task and the executed motoric command, rather than simply by directional isotropy. As suggested by Patel et al. (2016) and observed by Ning (2022b), it is possible that participants’ comfortable pitch may reside at the lower end of their vocal pitch range, making them more capable of raising their pitch rather than lowering it. Consequently, the larger *pitch-increasing* responses observed in non-steady pitch vocalizations in our study may be associated with the availability of vocal pitch range toward the high end, which participants can utilize. One caveat to note is that our participants produced glides that spanned roughly 4 semitones. It would be interesting to explore whether the directional effect exists when the gliding pitch is further expanded or approaches the extremes of available vocal pitch range.

One methodological difference between our study and Burnett and Larson (2002) is the timing of pitch-shifts, occurring either at the onset (in the former) or midway (in the latter) of vocalization. In the present study, the glissandos consisted of a 0.5 s steady note, a 2 s upward/downward glide, and a 0.5 s steady note, with pitch-shift stimuli appearing at the onset of the glide. In contrast, Burnett and Larson (2002) used the glissandos with a 1 s steady note, a 4 s upward glide, and a 1 s steady note, with pitch-shift stimuli occurring 2.5–3.5 s after vocal onset. The vocalization length was shortened in this study to reduce the difficulty for non-musicians. However, the timing of pitch-shift stimuli may influence the degree of compensation. Previous research has suggested that reduced susceptibility to pitch perturbations (i.e., diminished responses) may occur at the initial stage of speech planning (Liu et al., 2009; Xu et al., 2004), probably because recalculation for pitch could still be available at the beginning. Further research is needed to better understand the impact of stimulus timing in glissandos.

## 5 Conclusion

The present study investigated the roles of musical expertise and task-specificity on the proportions and magnitudes of pitch-shift responses, encompassing both opposing and following responses, under auditory perturbations. Musicians were less susceptible to pitch perturbations in comparison to non-musicians, resulting in reduced opposing and following responses in both sustained vowels and glissandos. The prevalence of substantial proportions of following responses on a trial-to-trial basis highlights that following responses should not be considered a minority within auditory perturbation responses. The occurrence of either opposing responses or following responses in individual trials is contingent upon the interaction between shift direction and intended pitch direction. Both opposing and following responses were significantly larger in the glissandos as compared to the sustained vowels, indicating that steady pitch and non-steady pitch regulation may involve distinct mechanisms. Overall, the results of this study suggest that our auditory-vocal control is contextually sensitive, and musical training plays a role in shaping how participants respond to auditory perturbations.

## Author’s note

Results from half of the participants (*N* = 14) were previously presented at the 20th International Congress of Phonetic Sciences (ICPhS 2023). This manuscript offers a comprehensive analysis of the entire sample (*N* = 34) and includes additional Bayesian statistical findings for the proportional data.

## Data Availability

The raw data supporting the conclusions of this article will be made available by the author, without undue reservation.

## Supplementary material

Supplementary Figure 1 Trace plots of the four chains from Markov chain Monte Carlo (MCMC) simulations across the four chains.

Supplementary Figure 2 Kernel density plots of parameter estimates obtained from the posterior distributions. Distributions that overlap zero indicate insignificance.
