# Supplementary figures and images for: The impact of musical expertise and directional isotropy on the proportions and magnitudes of pitch-shift responses in glissandos
Source: Front Psychol. 2025 Jan 23;15:1332028. doi: 10.3389/fpsyg.2024.1332028 (PMC11800587; doi:10.3389/fpsyg.2024.1332028)

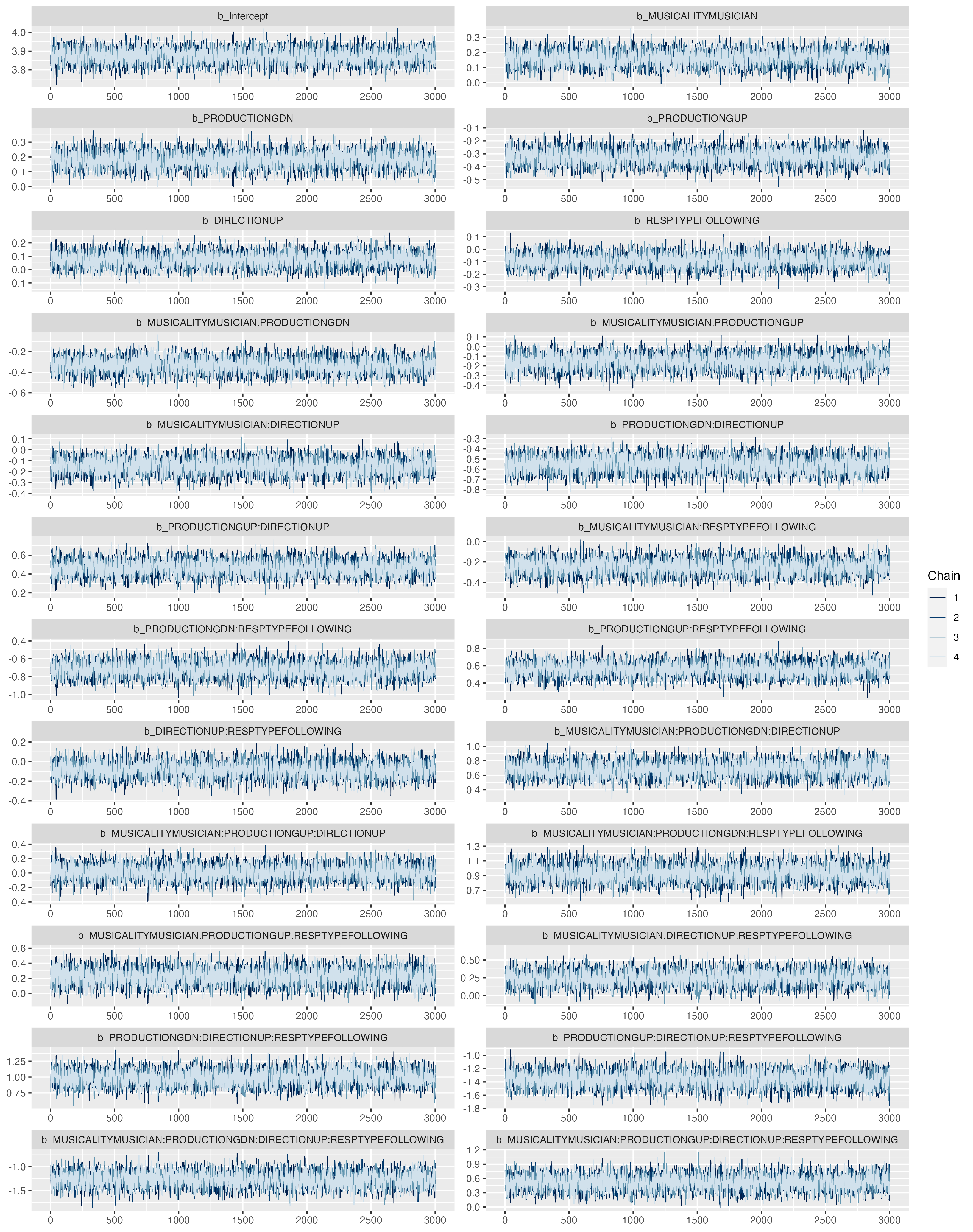

Supplement: Supplementary Figure 1 — Trace plots of the four chains from Markov chain Monte Carlo (MCMC) simulations across the four chains. [file Image_1.tiff]

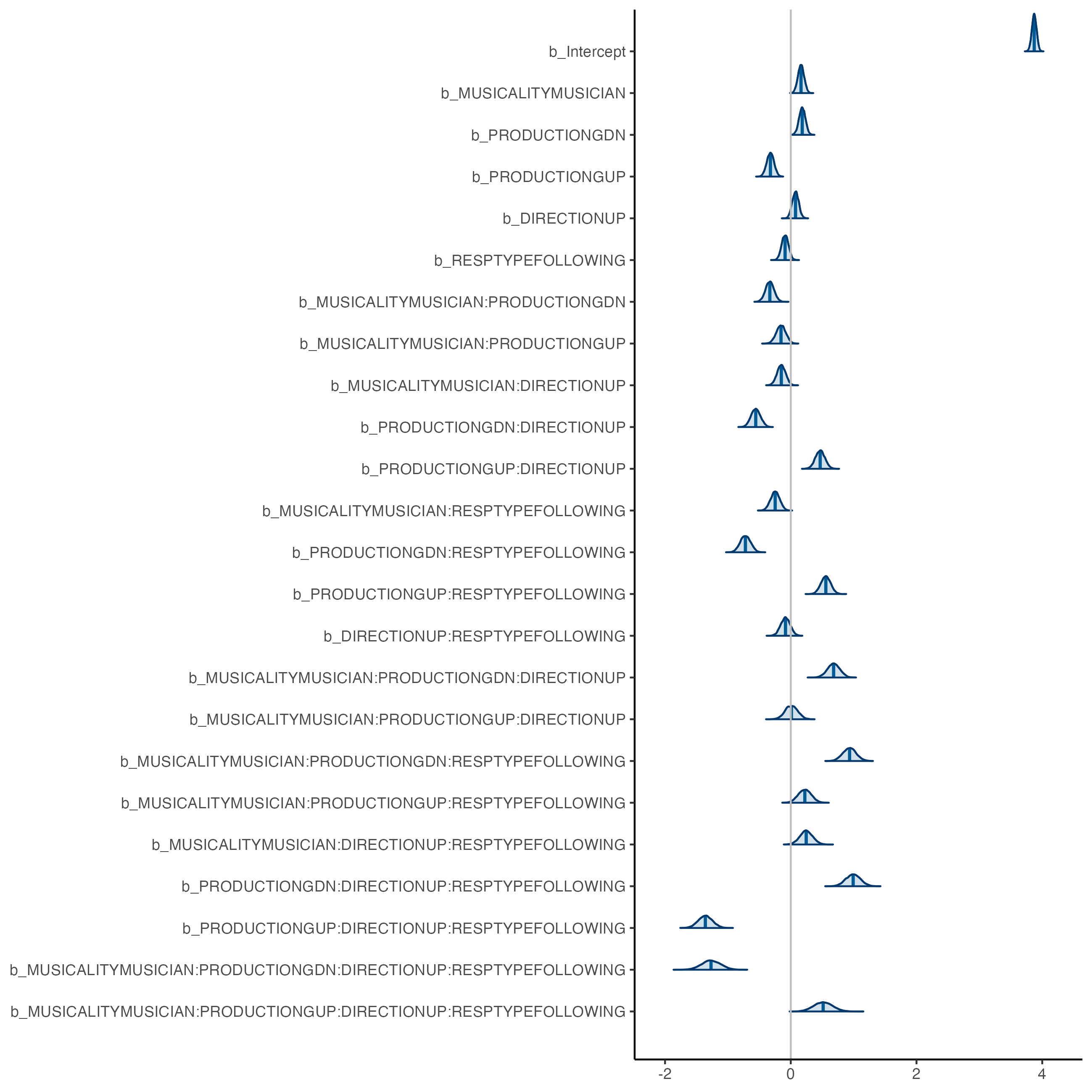

Supplement: Supplementary Figure 2 — Kernel density plots of parameter estimates obtained from the posterior distributions. Distributions that overlap zero indicate insignificance. [file Image_2.tiff]
